# Editorial: Benign prostatic hyperplasia and overactive bladder: new members of metabolic syndrome

**DOI:** 10.3389/fruro.2023.1272592

**Published:** 2023-08-25

**Authors:** Xiaolong Wang, Qingfeng Yu, Martin C. Michel

**Affiliations:** ^1^ Department of Urology, Zhongnan Hospital of Wuhan University, Wuhan, China; ^2^ Department of Pathology and Laboratory Medicine, Temple University, Philadelphia, PA, United States; ^3^ Department of Urology, The First Affiliated Hospital of Guangzhou Medical University, Guangzhou, China; ^4^ Department of Pharmacology, Johannes Gutenberg University, Mainz, Germany

**Keywords:** metabolic syndrome, prostate, hyperplasia, overactive bladder, lower urinary tract symptoms

Benign prostatic hyperplasia (BPH) and overactive bladder syndrome (OAB) represent highly prevalent conditions, particularly in the elderly that frequently culminate in bothersome lower urinary tract symptoms (LUTS) and reduced quality of life. The intricate pathophysiology underlying these disorders remains incompletely defined, with myriad contributory factors at play. Nevertheless, an accumulating body of evidence underscores previously unrecognized roles for metabolic aberrations linked to metabolic syndrome (MetS) and associated disorders such as diabetes or arterial hypertension as relevant factors linked to LUTS in BPH and OAB. This editorial synthesizes emerging clinical and preclinical data that unveil novel mechanistic connections between metabolic dysregulation and lower urinary tract dysfunction.

## Clinical correlations between MetS and LUTS severity

Within this aggregate of investigations, quantitative correlations have been established between individual components of MetS and exacerbated LUTS. Müderrisoglu et al. demonstrated associations of diabetes and hypertension with more pronounced baseline LUTS and attenuated therapeutic responses in OAB patients. Furthermore, Erdogan et al. discovered increased bladder weight, across five murine models exhibiting comorbid obesity and/or diabetes. These preclinical findings corroborate the putative role of metabolic abnormalities in aggravating LUTS.

## Broader systemic impacts on the cardiovascular system

Explorations of clinical data by Chan et al. revealed correlations between LUTS, especially nocturia, arterial stiffness, and major adverse cardiovascular events in males with MetS. This relationship implies broader systemic impacts of MetS on both the urinary and cardiovascular systems, potentially mediated by shared pathophysiological mechanisms. However, Michel et al. identified only a weak association between hypertension and baseline LUTS/treatment outcomes (similar to the work of Müderrisoglu et al.), underscoring the complexity of interactions between cardiovascular and metabolic components and LUTS pathogenesis.

## Dissecting the multifaceted mechanistic underpinnings

The cumulative evidence robustly indicates MetS likely promotes the development of LUTS linked to BPH and OAB via intricate, multifactorial pathways culminating in atherosclerosis and reduced tissue perfusion. Specific MetS characteristics, particularly obesity, diabetes, and arterial stiffness, appear strongly correlated with LUTS severity, emphasizing the need to define discrete molecular mechanisms to enable targeted therapeutic development.

## Hormonal imbalance as a contributing factor

Endocrine disturbances are cardinal features of MetS that may further drive LUTS pathogenesis. Wang et al. reported that imbalances in sex steroid and insulin-like growth factor signaling have been proposed to affect prostate overgrowth and bladder dysfunction. Disentangling the precise interplay between hormones, metabolic derangements, and urinary symptomatology could unveil innovative drug targets.

## Therapeutic opportunities to concurrently target MetS and LUTS

Recognition of the intricate associations between MetS pathologies and LUTS creates novel prospects for tailored therapeutic interventions. Lifestyle adjustments to diet and activity levels represent first-line management of MetS that could beneficially impact urinary dysfunction. Emerging drug candidates that concomitantly ameliorate metabolic abnormalities and LUTS may also hold promise. Additionally, personalized treatment regimens adapted to an individual’s metabolic profile could optimize outcomes.

## Concluding remarks

The recent body of evidence elucidating connections between MetS and LUTS associated with BPH and OAB has significantly advanced current comprehension of this intricate interplay. Quantitative correlations between discrete metabolic aberrations and exacerbated LUTS have been consistently demonstrated, underscoring the imperative to address metabolic factors in the management of these urologic conditions. Diverse patient cohorts and preclinical models have illuminated roles for insulin resistance, metabolic hormone disturbances, sex steroid imbalances, and chronic inflammation in the pathogenesis of MetS-related LUTS. Furthermore, modifiable lifestyle factors including dietary quality, physical activity, and smoking have emerged as major environmental contributors. Nevertheless, fully deciphering precise molecular mechanisms and causative relationships necessitates additional longitudinal clinical studies tracking MetS indices and LUTS over time, complemented by rigorously controlled animal models. By building on these research foundations, the development of innovative prevention and treatment strategies promises to dramatically improve quality of life for aging men encumbered by these burdensome urological disorders.

Building on the foundation laid by this compendium of research, future investigations should focus on identifying novel biomarkers for early detection and risk stratification of LUTS in the context of MetS. A deeper understanding of the genetic underpinnings of MetS-related LUTS could pave the way for personalized medicine, tailoring therapies based on individual metabolic profiles.

Moreover, collaborations between urologists, endocrinologists, cardiologists, and researchers from various disciplines are crucial to fully comprehend the intricate web of interactions between MetS and LUTS. These multidisciplinary efforts will accelerate the translation of scientific findings into clinically relevant applications, benefiting patients through improved diagnostics, treatment options, and patient care.

In addition, patient education and awareness programs must be emphasized to empower individuals to take an active role in managing their metabolic health. Addressing lifestyle factors, such as diet, physical activity, and stress management, can have a profound impact on both MetS and LUTS. Public health initiatives aimed at promoting healthier lifestyles should be encouraged to reduce the burden of MetS-related LUTS in aging populations.

As we make progress in better understanding the crosstalk between MetS and LUTS, it is imperative that we consider the broader societal impact. Healthcare policies and guidelines should be informed by this growing body of evidence to ensure equitable access to high-quality urological care for all individuals affected by MetS-related LUTS.

In conclusion, the convergence of scientific research in the fields of urology and metabolic disorders has shed light on the profound relationship between MetS and LUTS. Through concerted efforts, spanning from clinical investigations to preclinical studies and from patient education to precision medicine, we stand poised to transform the management of BPH and OAB-associated LUTS. By mitigating the encumbrance of these urological conditions through innovative prevention and treatment approaches, researchers can empower aging men to enjoy enhanced quality of life and improved health span, unfettered by the constraints imposed by MetS-related LUTS.

## Author contributions

XW: Writing – original draft. QY: Writing – review & editing. MM: Writing – review & editing.

